# One-year outcomes in cardiogenic shock triggered by ventricular arrhythmia: An analysis of the FRENSHOCK multicenter prospective registry

**DOI:** 10.3389/fcvm.2023.1092904

**Published:** 2023-01-26

**Authors:** Miloud Cherbi, François Roubille, Nicolas Lamblin, Laurent Bonello, Guillaume Leurent, Bruno Levy, Meyer Elbaz, Sebastien Champion, Pascal Lim, Francis Schneider, Alain Cariou, Hadi Khachab, Jeremy Bourenne, Marie-France Seronde, Guillaume Schurtz, Brahim Harbaoui, Gerald Vanzetto, Charlotte Quentin, Xavier Delabranche, Nadia Aissaoui, Nicolas Combaret, Danka Tomasevic, Benjamin Marchandot, Benoit Lattuca, Patrick Henry, Edouard Gerbaud, Eric Bonnefoy, Etienne Puymirat, Philippe Maury, Clément Delmas

**Affiliations:** ^1^Intensive Cardiac Care Unit, Rangueil University Hospital, Toulouse, France; ^2^Institute of Metabolic and Cardiovascular Diseases (I2MC), UMR-1048, National Institute of Health and Medical Research (INSERM), Toulouse, France; ^3^PhyMedExp, Université de Montpellier, INSERM, CNRS, Cardiology Department, INI-CRT, CHU de Montpellier, Montpellier, France; ^4^Department of Cardiology, Urgences et Soins Intensifs de Cardiologie, CHU Lille, University of Lille, Inserm U1167, Lille, France; ^5^Aix-Marseille Université, Marseille, France; ^6^Intensive Care Unit, Department of Cardiology, Assistance Publique-Hôpitaux de Marseille, Hôpital Nord, Marseille, France; ^7^Mediterranean Association for Research and Studies in Cardiology (MARS Cardio), Marseille, France; ^8^Department of Cardiology, CHU Rennes, Inserm, LTSI-UMR 1099, Univ Rennes 1, Rennes, France; ^9^CHRU Nancy, Réanimation Médicale Brabois, Nancy, France; ^10^Clinique de Parly 2, Ramsay Générale de Santé, Le Chesnay, France; ^11^Université Paris Est-Créteil, INSERM, IMRB, Créteil, France; ^12^AP-HP, Hôpital Universitaire Henri-Mondor, Service de Cardiologie, Créteil, France; ^13^Médecine Intensive-Réanimation, Hôpital de Hautepierre, Hôpitaux Universitaires de Strasbourg, Strasbourg, France; ^14^Medical Intensive Care Unit, Cochin Hospital, Assistance Publique-Hôpitaux de Paris, Centre–Université de Paris, Medical School, Paris, France; ^15^Intensive Cardiac Care Unit, Department of Cardiology, CH d’Aix-en-Provence, Aix-en-Provence, France; ^16^Aix-Marseille Université, Service de Réanimation des Urgences, CHU La Timone 2, Marseille, France; ^17^Servicede Cardiologie CHU Besançon, Besançon, France; ^18^Cardiology Department, Hôpital Croix-Rousse and Hôpital Lyon Sud, Hospices Civils de Lyon, Lyon, France; ^19^Department of Cardiology, University of Lyon, CREATIS UMR5220, INSERM U1044, INSA-15, Lyon, France; ^20^Department of Cardiology, Hôpital de Grenoble, Grenoble, France; ^21^Service de Réanimation Polyvalente, Centre Hospitalier Broussais, 1 Rue de la Marne, Saint-Malo, France; ^22^Réanimation Chirurgicale Polyvalente, Pôle Anesthésie–Réanimation Chirurgicale–Médecine Péri-opératoire, Les Hôpitaux Universitaires de Strasbourg, Nouvel Hôpital Civil 1, Porte de l’Hôpital, Strasbourg, France; ^23^Department of Cardiology, CHU Clermont-Ferrand, CNRS, Université Clermont Auvergne, Clermont-Ferrand, France; ^24^Intensive Cardiac Care Unit, Lyon Brom University Hospital, Lyon, France; ^25^Université de Strasbourg, Pôle d’Activité Médico-Chirurgicale Cardio-Vasculaire, Nouvel Hôpital Civil, Centre Hospitalier Universitaire, Strasbourg, France; ^26^Department of Cardiology, Nîmes University Hospital, University of Montpellier, Nîmes, France; ^27^Assistance Publique-Hôpitaux de Paris (AP-HP), Hôpital Lariboisière, Department of Cardiology, Paris, France; ^28^Intensive Cardiac Care Unit and Interventional Cardiology, Hôpital Cardiologique du Haut Lévêque, Pessac, France; ^29^Bordeaux Cardio-Thoracic Research Centre, U1045, Bordeaux University, Hôpital Xavier Arnozan, Pessac, France; ^30^Assistance Publique-Hôpitaux de Paris (AP-HP), Hôpital Européen Georges Pompidou, Department of Cardiology, Paris, France; ^31^Université de Paris, Paris, France; ^32^REICATRA, Institut Saint Jacques, CHU de Toulouse, Toulouse, France

**Keywords:** cardiogenic shock, ventricular tachycardia, ventricular arrhythmia, epidemiology, prognosis

## Abstract

**Background:**

Cardiogenic shock (CS) is a life-threatening condition carrying poor prognosis, potentially triggered by ventricular arrhythmia (VA). Whether the occurrence of VA as trigger of CS worsens the prognosis compared to non-VA triggers  remains  unclear.  The  aim  of  this  study  was  to  evaluate  1-year  outcomes [mortality, heart transplantation, ventricular assist devices (VAD)] between VA-triggered and non-VA-triggered CS.

**Methods:**

FRENSHOCK is a prospective multicenter registry including 772 CS patients from 49 centers. One to three triggers can be identified in the registry (ischemic, mechanical complications, ventricular/supraventricular arrhythmia, bradycardia, iatrogenesis, infection, non-compliance). Baseline characteristics, management and 1-year outcomes were analyzed according to the VA-trigger in the CS population.

**Results:**

Within 769 CS patients included, 94 were VA-triggered (12.2%) and were compared to others. At 1 year, although there was no mortality difference [42.6 vs. 45.3%, HR 0.94 (0.67–1.30), *p* = 0.7], VA-triggered CS resulted in more heart transplantations and VAD (17 vs. 9%, *p* = 0.02). Into VA-triggered CS group, though there was no 1-year mortality difference between ischemic and non-ischemic cardiomyopathies [42.5 vs. 42.6%, HR 0.97 (0.52–1.81), *p* = 0.92], non-ischemic cardiomyopathy led to more heart transplantations and VAD (25.9 vs. 5%, *p* = 0.02).

**Conclusion:**

VA-triggered CS did not show higher mortality compared to other triggers but resulted in more heart transplantation and VAD at 1 year, especially in non-ischemic cardiomyopathy, suggesting the need for earlier evaluation by advanced heart failure specialized team for a possible indication of mechanical circulatory support or heart transplantation.

**Clinical trial registration:**

https://clinicaltrials.gov, identifier NCT02703038.

## Graphical Abstract

The FRENSHOCK registry–One-year outcomes in cardiogenic shock triggered by ventricular arrhythmia. Underlying cardiopathy was considered ischemic in the presence of at least one culprit lesion hemodynamically significant on coronary angiography (stenosis or thrombosis). CS, cardiogenic shock; ICD, implantable cardioverter defibrillator; LVEF, left ventricular ejection fraction; VA, ventricular arrhythmia; VAD, ventricular assist device. 
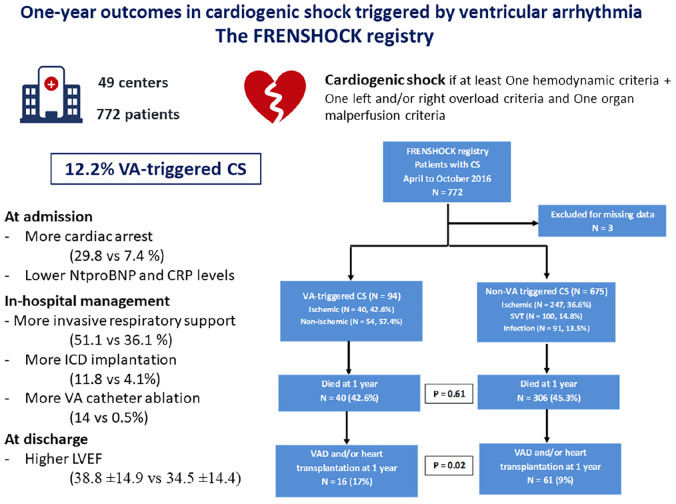

## Introduction

Cardiogenic shock (CS) is a life-threatening condition characterized by inadequate cardiac output. CS remains common in intensive cardiac care unit (ICCU) (1, 2), carrying a poor prognosis with a mortality rate of 25–35% at 1 month (3) and 45–60% at 1 year (4). Several prognostic factors for mortality have been established, including age, lactatemia at admission (5), renal replacement therapy, or use of catecholamines (4).

Acute coronary syndrome (ACS) remains the leading underlying heart disease (6, 7), and reduces both immediate and long-term survival in case of CS (8). By contrast, CS in non-ischemic cardiomyopathy is less studied raising concerns about different prognosis and specific management (2, 9).

Furthermore, the relationship between VA and heart failure is still under debate (10). Even if mounting evidence indicates that high VA burden seems closely linked to mortality and outcomes in many settings of chronic heart failure (11–13), their significance in the context of CS remains unclear: whether the VA-triggered CS results in worse long-term outcomes than the non-VA-triggered one is not established (14).

Hence, the aim of this study was to compare 1-year outcomes between VA-triggered CS and non-VA triggered CS, based on the multicenter prospective FRENSHOCK registry.

## Materials and methods

### Patient population

As previously described (15), FRENSHOCK is an observational, prospective, multicenter registry, including 772 patients admitted for CS between April and October 2016 in ICU/ICCU in France. All institutions were invited to participate, including university hospitals, general and regional hospitals, public and private hospitals (ICCUs, surgical ICUs, medical ICUs, and general ICUs).

All adult patients (≥18 years old) with CS were prospectively included in this registry if they met at least one criterion of each of the following three components: (1) Low cardiac output: low SBP < 90 mmHg and/or the need for maintenance with vasopressors/inotropes and/or a low cardiac index < 2.2 L/min/m^2^; (2) Left and/or right heart filling pressure elevation, defined by clinical signs, radiology, blood tests, echocardiography, or signs of invasive hemodynamic overload and (3) Signs of organ malperfusion, which could be clinical (oliguria, confusion, pale and/or cold extremities, mottled skin) and/or biological (lactate > 2 mmol/L, metabolic acidosis, renal failure, liver insufficiency).

For each patient, investigators were invited to identify one to three triggers among the following: ischemic (type 1 or 2 AMI), mechanical complications (valvular injury, ventricular septal defect), ventricular and supraventricular arrhythmia, severe bradycardia, iatrogenesis (medication induced), infections, non-observance of previous medication. Underlying cardiopathy was considered ischemic in the presence of at least one culprit lesion hemodynamically significant on coronary angiography (stenosis, thrombosis). VA-triggered CS status was defined by the managing physician.

### Data collection

First, general data on cardiological history (heart disease, previous ICD), coexisting conditions (kidney or pulmonary disease, cancer), risk factors (smoking status, hypertension, dyslipidemia, diabetes mellitus), treatments (including antiarrhythmic drugs) were recorded. Clinical, biological and echocardiographic data were collected at admission and 24 h. Clinical assessment included blood pressure, heart rate, sinus rhythm, signs of left and/or right heart failure, mottling, cardiac arrest. Biological data included bilirubin and creatinine levels, serum electrolytes, prothrombin time, hemoglobin, arterial blood gases and arterial lactate, C-reactive protein, troponin, BNP/Nt-proBNP. Echocardiographic evaluation mandatorily included left ventricular ejection fraction (visual evaluation or biplane Simpson’s method), presence of pericardial effusion and severe valvulopathy (defined as grade IV), in addition to which parameters such as TAPSE or S wave were often described.

Data on CS management included pharmacological treatment at admission, at discharge and at 1 year (catecholamines, beta-blockers, diuretics, ACEi, ARB, MRA, sacubitril/valsartan, antiarrhythmic), organ replacement therapies such as mechanical ventilation (invasive and/or non-invasive), short-term circulatory support (IABP, extracorporeal membrane oxygenation, Impella^®^) and renal replacement therapy.

### Outcomes

Short and long-term outcomes, including all-cause mortality, heart transplantation or ventricular assist devices (VAD), were assessed at 1 month and 1 year. The primary end point was 1-year all-cause mortality. Secondary end points included 1-month all-cause mortality, need for heart transplantation or VAD, rate of rehospitalizations, and the composite of death, heart transplantation or VAD. We investigated the cause of death in the VA group, distinguishing four possibilities (end-stage heart failure, sudden cardiac death or recurrence of intractable VA, other, unknown). Further comparisons were made between ischemic and non-ischemic cardiomyopathy, as well as between patients presenting with acute and chronic coronary syndromes. When done, VA catheter ablation (16) and myocardial revascularization (17) were performed according to the current techniques.

### Ethics

The study was conducted in accordance with guidelines for good clinical practice and French law. Written consent was obtained for all patients. Recorded data and their storage were approved by the CCTIRS (French Health Research Data Processing Advisory Committee) (no 15.897) and the CNIL (French Data Protection Agency) (no DR-2016-109).

### Statistical analysis

Continuous variables are reported as means (SD) or medians and interquartile ranges (IQR) when appropriate. Categorical variables are described in numbers and percentages. Comparisons were made using Mann Whitney non-parametric test for continuous variables and chi-square test or Fisher’s exact test for categorical variables. All-cause mortality was assessed using Kaplan-Meier curves, and Cox proportional hazards models were used to determine the HR and 95% confidence interval (CI) for mortality. Log-rank test was carried out to compare survival between groups. An additional propensity score matching analysis was performed with the greedy nearest neighbor algorithm (6:1 ratio) using a multivariable logistic regression model including five covariates (age, sex, history of ischemic heart disease, LVEF ≤ 40% at admission, previous ICD) that were prognostically important for the outcome and to minimize confounding factors. A second comparison was made in VA-triggered CS group between ischemic and non-ischemic VA. Analysis were performed using R software [version 4.1.2 (2021-11-01)]. A *p* value < 0.05 was considered statistically significant.

## Results

### Overall population

Seven hundred seventy-two patients with CS were included in 49 centers, of which 3 were excluded for missing data (Graphical Abstract). Among the 769 patients, 94 were VA-triggered (12.2%). Table 1 summarizes baseline characteristics. Mean age was 65.8 ± 14.8 years, with a predominance of men (71.4%). 56% were already known for previous cardiac history (29.9% ischemic and 1% dilated) and cardiovascular risk factors were frequent (respectively, 47.3, 36.1, 28.3, and 27.8% for hypertension, dyslipidemia, diabetes, and current smoking). Ongoing long-term therapies for previous heart failure were common at admission (respectively, 41.1, 37.9, and 13.8% for betablockers, ACEi/ARB and aldosterone antagonist). Long-term antiarrhythmic drug therapies (essentially amiodarone) were noted in 17.4% of them.

**TABLE 1:** Baseline characteristics at admission according to cardiogenic shock triggers (VA versus non-VA).

	Overall population (*n* = 769)	VA-triggered CS (*n* = 94)	Non-VA triggered CS (*n* = 675)	*P*-value
Age, mean ± SD, years	65.8 ± 14.8	64.1 ± 14.5	66.0 ± 14.9	0.25
Male, *n* (%)	549 (71.4)	62 (66.0)	487 (72.1)	0.21
Body mass index, mean ± SD, kg/m^2^	25.9 ± 5.5 (*n* = 741)	25.8 ± 4.6 (*n* = 88)	25.9 ± 5.7 (*n* = 653)	0.56
**Risk factors, *n* (%)**				
Diabetes mellitus	217 (28.3) (*n* = 767)	23 (24.5)	194 (28.8) (*n* = 673)	0.38
Hypertension	363 (47.3) (*n* = 768)	38 (40.4)	325 (48.2) (*n* = 674)	0.16
Dyslipidemia	277 (36.1) (*n* = 768)	38 (40.4)	239 (35.5) (*n* = 674)	0.38
Current smoker	205 (27.8) (*n* = 737)	29 (31.9) (*n* = 91)	176 (27.2) (*n* = 646)	0.36
**Medical history, *n* (%)**				
Peripheral artery disease	91 (11.8) (*n* = 768)	8 (8.5)	83 (12.3) (*n* = 674)	0.29
Myocardial revascularization	203 (26.4) (*n* = 768)	21 (22.3)	182 (27.0) (*n* = 674)	0.34
Chronic kidney disease	163 (21.2) (*n* = 768)	13 (13.8)	150 (22.3) (*n* = 674)	0.06
ICD	127 (16.5) (*n* = 768)	14 (14.9)	113 (16.8) (*n* = 674)	0.65
COPD	50 (6.5) (*n* = 768)	4 (4.3)	46 (6.8) (*n* = 674)	0.34
Active cancer	51 (6.6) (*n* = 768)	5 (5.3)	46 (6.8) (*n* = 674)	0.58
Stroke	62 (8.1) (*n* = 768)	9 (9.6)	53 (7.9) (*n* = 674)	0.57
**History of cardiac disease, *n* (%)**				
All causes	430 (56.0) (*n* = 768)	47 (50.0)	383 (56.8) (*n* = 674)	0.26
Ischemic	230 (29.9) (*n* = 768)	24 (25.5)	206 (30.6) (*n* = 674)	
Hypertrophic	11 (1.4) (*n* = 768)	1 (1.1)	10 (1.5) (*n* = 674)	
Toxic	33 (4.3) (*n* = 768)	4 (4.3)	29 (4.3) (*n* = 674)	0.71
Dilated	77 (1.0) (*n* = 768)	12 (12.8)	65 (9.6) (*n* = 674)	
**Previous medications, *n* (%)**				
Aspirin	288 (37.5) (*n* = 767)	37 (39.4)	251 (37.3) (*n* = 673)	0.78
P2Y12 inhibitors	126 (16.4) (*n* = 767)	20 (21.3)	106 (15.8) (*n* = 673)	0.23
Vitamin K antagonist	163 (21.3) (*n* = 767)	12 (12.8)	151 (22.4) (*n* = 673)	0.04
Direct oral anticoagulant	56 (7.3) (*n* = 767)	5 (5.3)	51 (7.6) (*n* = 673)	0.56
ACEi or ARB	291 (37.9) (*n* = 767)	33 (35.1)	258 (38.3) (*n* = 673)	0.62
Sacubitril/valsartan	17 (2.3) (*n* = 742)	1 (1.1) (*n* = 91)	16 (2.5) (*n* = 651)	0.71
Statins	286 (37.3) (*n* = 767)	38 (40.4)	248 (36.8) (*n* = 673)	0.58
Beta blockers	315 (41.1) (*n* = 767)	38 (40.4)	277 (41.2) (*n* = 673)	0.98
Loop diuretics	373 (48.6) (*n* = 767)	40 (42.6)	333 (49.5) (*n* = 673)	0.25
Aldosterone antagonist	106 (13.8) (*n* = 767)	10 (10.6)	96 (14.3) (*n* = 673)	0.43
Thiazide diuretics	44 (5.9) (*n* = 751)	6 (6.5) (*n* = 93)	38 (5.8) (*n* = 658)	0.98
Non-dihydropyridine CCB	16 (2.2) (*n* = 731)	0 (0) (*n* = 93)	16 (2.5) (*n* = 638)	0.25
Amiodarone	130 (17.4) (*n* = 749)	21 (22.6) (*n* = 93)	109 (16.6) (*n* = 656)	0.2
Other antiarrhythmic	30 (4.0) (*n* = 743)	7 (7.5) (*n* = 93)	23 (3.5) (*n* = 650)	0.09

ACEi, angiotensin-converting enzyme inhibitor; ARB, angiotensin receptor blocker; CCB, calcium channel blocker; COPD, chronic obstructive pulmonary disease; CS, cardiogenic shock; ICD, implantable cardiac defibrillator; SD, standard deviation; VA, ventricular arrhythmia.

As reported in Table 2, mean SBP was 101.3 ± 25.2 mmHg, with mottling in 39%. Initial echocardiographic data revealed mean LVEF of 26.3 ± 13.4%, median TAPSE of 13 mm (10–16) and median PSVtdi of 8 cm/s (6–11).

**TABLE 2:** Clinical, echocardiographic, and biological parameters at admission according to cardiogenic shock triggers (VA vs. non-VA).

	Overall population (*n* = 769)	VA-triggered CS (*n* = 94)	Non-VA triggered CS (*n* = 675)	*P*-value
**Clinical presentation at admission**				
Heart rate, mean ± SD, bpm	95.7 ± 29.6 (*n* = 766)	102.0 ± 42.5	94.9 ± 27.2 (*n* = 672)	0.43
SBP, mean ± SD, mmHg	101.3 ± 25.2 (*n* = 767)	98.8 ± 23.6	101.6 ± 25.4 (*n* = 673)	0.63
DBP, mean ± SD, mmHg	63.2 ± 17.4 (*n* = 766)	61.2 ± 17.0	63.5 ± 17.5 (*n* = 672)	0.39
MBP, mean ± SD, mmHg	74.9 ± 18.4 (*n* = 764)	73.2 ± 18.8	75.2 ± 18.3 (*n* = 670)	0.74
Sinus rhythm, *n* (%)	398 (52.0) (*n* = 765)	32 (34.0)	366 (54.6) (*n* = 671)	<0.01
Mottling, *n* (%)	256 (39.0) (*n* = 657)	38 (47.5) (*n* = 80)	218 (37.8) (*n* = 577)	0.12
Cardiac arrest, *n* (%)	78 (10.2) (*n* = 768)	28 (29.8)	50 (7.4) (*n* = 674)	<0.01
**Blood tests at admission, median (IQR)**				
Sodium, mmol/L	135 (132–139) (*n* = 757)	135 (133–139) (*n* = 93)	135 (131–139) (*n* = 664)	0.19
Creatinin, μmol/L	133 (96–189.5) (*n* = 758)	121 (92–177) (*n* = 93)	134 (97–193) (*n* = 665)	0.17
Bilirubin, mg/L	16 (9–29) (*n* = 541)	14 (9–22) (*n* = 77)	17 (9–30) (*n* = 464)	0.24
Hemoglobin, g/dL	12.6 (11–14) (*n* = 751)	13 (11–14) (*n* = 92)	12.4 (11–14) (*n* = 659)	0.23
Arterial blood lactates, mmol/L	3 (2–4.8) (*n* = 681)	2.72 (2–6) (*n* = 90)	3 (2–4.5) (*n* = 591)	0.62
ASAT, UI/L	90 (39–298.8) (*n* = 544)	131 (44.8–379.8) (*n* = 70)	86 (38–287.8) (*n* = 474)	0.09
ALAT, UI/L	59.5 (27–182.2) (*n* = 556)	92 (37.8–288.5) (*n* = 72)	57 (26–170.3) (*n* = 484)	0.03
PT,%	59 (37–77) (*n* = 728)	64 (38.3–76) (*n* = 90)	58 (37–77) (*n* = 638)	0.23
Nt-proBNP, pg/mL	9,516 (4,064 – 22,149) (*n* = 221)	5,360 (724 – 10,592) (*n* = 37)	10,763 (4,532 – 25,222) (*n* = 184)	<0.01
BNP, pg/mL	1,150 (476.8 – 2,757.3) (*n* = 264)	660 (269.5 – 1,966) (*n* = 27)	1175 (509 – 2,834) (*n* = 237)	0.06
CRP, mg/L	28 (9–69.3) (*n* = 404)	15 (4–45) (*n* = 45)	29 (11–71) (*n* = 359)	<0.01
**Baseline echocardiography**				
LVEF, mean ± SD,%	26.3 ± 13.4 (*n* = 760)	24.4 ± 13.1 (*n* = 93)	26.6 ± 13.4 (*n* = 667)	0.11
TAPSE, median (IQR), mm	13 (10–16) (*n* = 257)	14 (10–17) (*n* = 32)	12 (10–16) (*n* = 225)	0.58
PSVtdi, median (IQR), cm/s	8 (6–11) (*n* = 205)	9 (6.8–12.3) (*n* = 24)	8 (6–10) (*n* = 181)	0.28
Severe mitral regurgitation, *n* (%)	106 (14.5) (*n* = 730)	10 (11.5) (*n* = 87)	96 (14.9) (*n* = 643)	0.49
Severe aortic stenosis, *n* (%)	36 (4.8) (*n* = 756)	1 (1.1) (*n* = 92)	35 (5.3) (*n* = 664)	0.11
Severe aortic regurgitation, *n* (%)	10 (1.3) (*n* = 752)	2 (2.2) (*n* = 92)	8 (1.2) (*n* = 660)	0.35

ALAT, alanine aminotransferase; ASAT, aspartate aminotransferase; BNP, Brain natriuretic peptide; CRP, C-reactive protein; CS, cardiogenic shock; DBP, diastolic blood pressure; IQR, interquartile range; MBP, mean blood pressure; Nt-proBNP, N-terminal-pro hormone BNP; LVEF, left ventricular ejection fraction; PSVtdi, peak systolic velocity tissue Doppler imaging; PT, prothrombin time; SBP, systolic blood pressure; SD, standard deviation; TAPSE, tricuspid annular plane systolic excursion; VA, ventricular arrhythmia.

### CS presentation and evolution at 24 h according to VA and non-VA groups

At admission, VA and non-VA triggered CS groups were similar regarding to age, sex, medical history or ongoing medication (Table 1). Only vitamin K antagonist was more frequent in the non-VA group (22.4 vs. 12.8%, p = 0.04).

Among the 675 non-VA triggered CS patients, main additional triggers were ischemic (32%), supra-ventricular tachycardia (14.8%), and infections (13.5%). By contrast, among the 94 VA-triggered CS patients, other most frequently associated triggers were ischemia (42.6%), mechanical complications (5.3%) and conduction disorders (5.3%) without statistical significance (Supplementary Table 1).

Clinical presentation was similar between groups at admission (Table 2) and 24 h (Supplementary Table 2) except a higher rate of cardiac arrest and a lower rate of sinus rhythm in the VA-triggered group (respectively, 29.8% vs. 7.4%, *p* < 0.01 and 34 vs. 54.6%, *p* < 0.01). Non-VA triggered CS patients presented with higher initial Nt-proBNP and CRP (respectively, 10,763 vs. 5,360 pg/ml, *p* < 0.01 and 29 vs. 15 mg/L, *p* < 0.01), higher creatinin and poorer prothrombin time at 24 h (respectively, 129 vs. 106 μmol/L, *p* = 0.04 and 60 vs. 70%, *p* < 0.01).

Echocardiographic evaluation showed similar biventricular dysfunction between groups with 24.4% vs. 26.6% (*p* = 0.11) and 14.0 vs. 12.0 mm (*p* = 0.58) for LVEF and TAPSE, respectively, for VA and non-VA triggered groups.

### In hospital management according to VA and non-VA groups

Table 3 summarizes in hospital management. Inotropes were used in 89.8%, without difference between VA and non-VA groups (respectively, 86.2 vs. 90.3%, *p* = 0.21). Dobutamine was the most frequently used (82.2% overall, 76.6 vs. 83.0%, *p* = 0.13), whereas norephinephrine was given in 53.5% (60.6 vs. 52.5%, *p* = 0.14) and levosimendan in 7.5% (5.3 vs. 7.7%, *p* = 0.4). Invasive ventilation was more frequently needed for VA-triggered CS (51.1 vs. 36.1%, p < 0.01). Short-term mechanical circulatory support needs were similar between groups for all categories, with 7.5 vs. 6.1% (*p* = 0.78) for IABP, 2.1 vs. 3.6% (*p* = 0.76) for Impella^®^ and 17 vs. 10.1% (*p* = 0.07) for ECLS. Renal replacement therapy was also equally used in the two groups (13.8 vs. 16.2%, *p* = 0.67).

**TABLE 3:** In-hospital management, short and long-term outcomes according to cardiogenic shock triggers (VA vs. non-VA).

	Overall population (*n* = 769)	VA-triggered CS (*n* = 94)	Non-VA triggered CS (*n* = 675)	*P*-value
**Medications used, *n* (%)**				
Dobutamine or norepinephrine or levosimendan	687 (89.8) (*n* = 765)	81 (86.2)	606 (90.3) (*n* = 671)	0.21
Dobutamine	629 (82.2) (*n* = 765)	72 (76.6)	557 (83.0) (*n* = 671)	0.13
Norepinephrine	409 (53.5) (*n* = 765)	57 (60.6)	352 (52.5) (*n* = 671)	0.14
Levosimendan	57 (7.5) (*n* = 765)	5 (5.3)	52 (7.7) (*n* = 671)	0.4
**Respiratory support, *n* (%)**				
Non-invasive	199 (26.0) (*n* = 765)	24 (25.5)	175 (26.1) (*n* = 671)	1
Invasive	290 (37.9) (*n* = 765)	48 (51.1)	242 (36.1) (*n* = 671)	<0.01
**Short-term mechanical circulatory support, *n* (%)**				
IABP	48 (6.3) (*n* = 765)	7 (7.5)	41 (6.1) (*n* = 671)	0.78
Impella^®^	26 (3.4) (*n* = 765)	2 (2.1)	24 (3.6) (*n* = 671)	0.76
ECLS	84 (11.0) (*n* = 766)	16 (17.0)	68 (10.1) (*n* = 672)	0.07
Renal replacement therapy, *n* (%)	122 (15.9) (*n* = 768)	13 (13.8)	109 (16.2) (*n* = 674)	0.67
LVEF at discharge, mean ± SD	35.0 ± 14.5 (*n* = 438)	38.8 ± 14.9 (*n* = 52)	34.5 ± 14.4 (*n* = 386)	0.04
**Mortality, *n* (%)**				
1 month	199 (25.9)	25 (26.6)	174 (25.8)	0.87
1 year	346 (45.0)	40 (42.6)	306 (45.3)	0.61
Rehospitalizations at 1 year, *n* (%)	308 (45.0) (*n* = 685)	41 (47.7) (*n* = 86)	267 (44.6) (*n* = 599)	0.59
Mortality or rehospitalizations at 1 year, *n* (%)	578 (84.4) (*n* = 685)	71 (82.6) (*n* = 86)	507 (84.6) (*n* = 599)	0.62
Heart transplantation or VAD at 1 year, *n* (%)	77 (10.0)	16 (17.0)	61 (9.0)	0.02
Mortality or heart transplantation or VAD at 1 year, *n* (%)	402 (52.3)	53 (56.4)	349 (51.7)	0.46

CS, cardiogenic shock; ECLS, extracorporeal life support; IABP, intra-aortic balloon pump; LVEF, left ventricle ejection fraction; SD, standard deviation; VA, ventricular arrhythmia; VAD, ventricular assist device.

### Antiarrhythmic therapy

All data related to anti-arrhythmic management are reported in Table 4. During initial care, data revealed similar use for all antiarrhythmic drugs, including amiodarone, betablockers and class 1 antiarrhythmic.

**TABLE 4:** Antiarrhythmic therapies according to cardiogenic shock triggers (VA vs. non-VA).

	Overall population (*n* = 769)	VA-triggered CS (*n* = 94)	Non-VA triggered CS (*n* = 675)	*P*-value
**Betablockers, *n* (%)**				
Initial care	315 (41.1) (*n* = 767)	38 (40.4)	277 (41.2) (*n* = 673)	0.98
24 h	95 (13.8) (*n* = 690)	15 (18.1) (*n* = 83)	80 (13.2) (*n* = 607)	0.23
Discharge	306 (56.0) (*n* = 546)	37 (56.9) (*n* = 65)	269 (55.9) (*n* = 481)	0.98
1 year	235 (65.1) (*n* = 361)	29 (69.1) (*n* = 42)	206 (64.6) (*n* = 319)	0.69
**Non-dihydropyridine CCB, *n* (%)**				
Initial care	16 (2.1) (*n* = 750)	0 (0) (*n* = 93)	16 (2.4) (*n* = 657)	0.24
24 h	5 (0.7) (*n* = 672)	1 (1.3) (*n* = 79)	4 (0.7) (*n* = 593)	0.47
Discharge	6 (1.1) (*n* = 529)	0 (0) (*n* = 62)	6 (1.3) (*n* = 467)	1
1 year	7 (2.1) (*n* = 331)	0 (0) (*n* = 40)	7 (2.4) (*n* = 291)	1
**Amiodarone, *n* (%)**				
Initial care	130 (17.4) (*n* = 749)	21 (22.3) (*n* = 93)	109 (16.6) (*n* = 656)	0.20
24 h	228 (33.4) (*n* = 682)	42 (51.9) (*n* = 81)	186 (30.9) (*n* = 601)	<0.01
Discharge	137 (25.8) (*n* = 531)	14 (22.6) (*n* = 62)	123 (26.2) (*n* = 469)	0.64
1 year	57 (17.2) (*n* = 331)	3 (7.5) (*n* = 40)	54 (18.6) (*n* = 291)	0.13
**Other anti-arrhythmic, *n* (%)**				
Initial care	30 (4.0) (*n* = 743)	7 (7.5) (*n* = 93)	23 (3.5) (*n* = 650)	0.09
24 h	53 (7.8) (*n* = 676)	12 (15) (*n* = 80)	41 (6.9) (*n* = 596)	0.02
Discharge	25 (4.7) (*n* = 534)	6 (9.5) (*n* = 63)	19 (4.0) (*n* = 471)	0.10
1 year	36 (10.9) (*n* = 329)	4 (10) (*n* = 40)	32 (11.1) (*n* = 289)	1
ICD implantation, *n* (%)	37 (5.1) (*n* = 731)	11 (11.8) (*n* = 93)	26 (4.1) (*n* = 638)	< 0.01
VAcatheter ablation, *n* (%)	16 (2.2) (*n* = 731)	13 (14.0) (*n* = 93)	3 (0.5) (*n* = 638)	< 0.01

CCB, calcium channel blocker; CS, cardiogenic shock; ICD, Implantable cardioverter-defibrillator; VA, ventricular arrhythmia.

Twenty-four hours after admission, amiodarone (51.9 vs. 30.9%, *p* < 0.01) and others antiarrhythmic drugs (class 1 or sotalol) (15 vs. 6.9%, *p* = 0.02) were more frequently used in the VA group, unlike betablockers (18.1 vs. 13.2%, *p* = 0.23).

At discharge as at 1 year, no difference was shown about the use of any antiarrhythmic drug.

An ICD had been previously implanted in 14.9% for the VA group and 16.8% for the non-VA group (*p* = 0.65). Within 1 year after CS, ICD implantation was required for 11.8% of the VA-group against 4.1% for the non-VA group (p < 0.01). VA catheter ablation was performed for 13 patients of the VA group and 3 of the non-VA group because of occurrence of VA after admission in this group (14 vs. 0.5%, *p* < 0.01).

### Short and long-term outcomes

**FIGURE 1:**
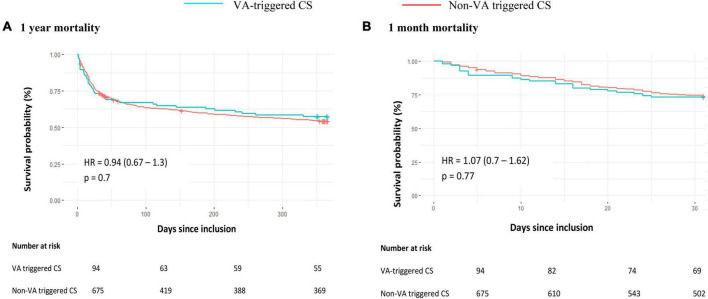
All-cause mortality in patients with cardiogenic shock according to a ventricular arrhythmia trigger at 1 year **(A)** and 1 month **(B)**. The cumulative incidences of 1-year and 1-month mortality were estimated with the use of the Kaplan–Meier method; hazard ratios and 95% confidence intervals were estimated with the use of Cox regression models. CS, cardiogenic shock; HR, hazard ratio; SVT, supra-ventricular tachycardia; VA, ventricular arrhythmia.

The Table 3 and Figure 1 show the absence of between-group difference in early or long-term all-cause mortality (26.6 vs. 25.8% and 42.6 vs. 45.3%, respectively, at 1-month and 1-year for VA and non-VA groups) nor in terms of 1-year rehospitalization (47.7 vs. 44.6% for VA and non-VA groups). LVEF at discharge was lower in the non-VA group (34.5 vs. 38.8%, *p* = 0.04) but VA-triggered CS resulted in more heart transplantation or need for VAD at one year compared to non-VA (17 vs. 9%, *p* = 0.02) (Table 3). The matched cohort included 658 patients (respectively, 94 and 564 for VA- and non-VA groups), with good balance between groups [all standardized mean differences below 0.1 after matching (Supplementary Tables 3, 4)], and did not show mortality difference, neither at 1 month [26.6 vs. 25.2%, HR 0.91 (95% CI 0.72–1.68), *p* = 0.67], nor at 1 year [42.6% vs. 44%, HR 0.98 (95% CI 0.70–1.37), *p* = 0.89] (Supplementary Figure 1).

### VA-triggered cardiogenic shocks

Among the 94 VA-triggered CS, 40 (42.6%) revealed at least one unknown culprit lesion on coronary angiography, respectively, located on LAD, RCA, LMCA, and LCX for 20 (50%), 10 (25%), 7 (17.5%), and 1 (2.5%) (2 unknown). Culprit lesion’s revascularization was performed for 36 (90%). At baseline, history of ICD implantation (25.9 vs. 0%, *p* < 0.01) and betablockers (53.7 vs. 22.5%, *p* < 0.01) were more frequently encountered for non-ischemic VA-triggered CS. Other baseline characteristics were similarly distributed (Supplementary Table 5).

While clinical and echocardiographic parameters were similar, biological presentation of the non-ischemic VA triggered CS appeared worse, with higher median levels of creatinine (138 vs. 115 μmol/L, *p* = 0.03), bilirubin (21 vs. 12, *p* < 0.01) and Nt-proBNP (6,787 vs. 1,520 pg/ml, *p* = 0.046) and lower PT (53.5 vs. 71%, *p* < 0.01) (Supplementary Table 5). All clinical, biological and echocardiographic data 24 h after admission are reported in Supplementary Table 6.

**FIGURE 2:**
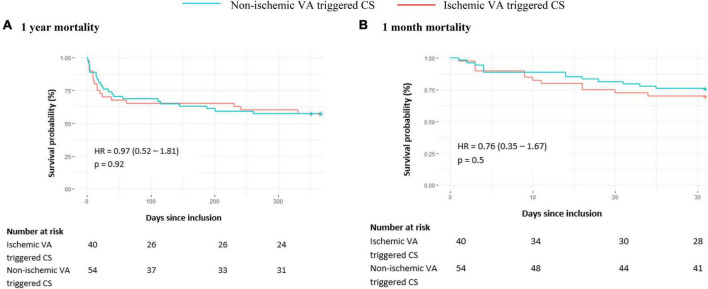
All-cause mortality in the VA group according to associated ischemic cardiomyopathy at 1 year **(A)** and 1 month **(B)**. The cumulative incidences of 1-year and 1-month mortality were estimated with the use of the Kaplan–Meier method; hazard ratios and 95% confidence intervals were estimated with the use of Cox regression models. Underlying cardiopathy was considered ischemic in the presence of at least one culprit lesion hemodynamically significant on coronary angiography (stenosis or thrombosis). CS, cardiogenic shock; HR, hazard ratio; VA, ventricular arrhythmia.

Survival analyses did not show difference of all-cause mortality at 1 month [30% vs. 24.1%, HR 0.76 (95% CI 0.35–1.67), *p* = 0.5] and 1 year [42.5 vs. 42.6%, HR 0.97 (95% CI 0.52–1.81), *p* = 0.92] (Figure 2) between ischemic and non-ischemic groups. At 1 year, heart transplantation or VAD were needed for 14 patients (25.9%) of the non-ischemic group versus 2 (5%) of the ischemic group (*p* = 0.02) (Supplementary Table 7). 1-year rehospitalizations rate was similar (45.9 vs. 49.0%, *p* = 0.95).

All data relating to in-hospital management and antiarrhythmic drugs are reported in Supplementary Tables 7, 8.

Among the 94 patients of the VA-group, 40 died at 1 year: death cause was available for 30 of them. 15 (50%) died because of end-stage heart failure, 11 (36.7%) because of other life-threatening conditions (sepsis, neoplasia, etc.) and 3 (13.3%) due to sudden cardiac death or recurrence of intractable VA.

Additional analyses showed no difference in 1-month or 1-year all-cause mortality when VA triggered CS patients with acute ischemia were compared to remaining patients with stable ischemic heart disease (Supplementary Figure 2). However, there was a trend toward a poorer outcome in patients with acute ischemia defined by elevated troponin (with a threshold value of 10 μUI/L for standard troponin I, 200 μg/L for high sensitivity troponin I, and 2,000 ng/mL for high sensitivity troponin T) even if it did not reach statistical significance (Supplementary Figure 3 and Supplementary Table 9).

## Discussion

To the best of our knowledge, FRENSHOCK is the largest European prospective, observational, multicenter registry on CS, representing a real-world cohort from a broad spectrum of etiologies. Even though VA is a well-known trigger of CS, only few studies have compared their long-term outcomes to other triggers. To date, this is the largest series analyzing such a great number of CS managed in routine practice and enabling to distinguish VA-triggered CS from others.

Primary endpoint of 1-year all-cause mortality did not show any difference between VA and non-VA triggered CS.

This lack of difference could be linked to the poor outcomes, regardless the cause of CS (global all cause-mortality at 1-year of 42–44%). It might be also explained by many confounding factors which cannot be corrected in this registry. First, it was not certain if VA-triggered CS represented a homogeneous population (CS truly triggered by VA or bystander VA in presence of CS induced by other causes): if the second situation was frequent, then the lack of difference is not surprising. Second, the information of electrical storm as a trigger for CS was not available in this registry: since such patients should have been aggressively treated either by ablation or efficient medical therapy, it is thus also not surprising that outcome is not poorer, since electrical storm does not convey higher mortality when successfully treated (18, 19).

In the current study, VA-triggered CS resulted in more heart transplantation and VAD. Similarly, into the VA-triggered CS group, although no all-cause mortality difference was shown between ischemic and non-ischemic cardiomyopathies, underlying non-ischemic heart disease resulted in more heart transplantation and ventricular assist devices.

Several hypotheses can be formulated to explain this trend. First, we hypothesized that patients from the VA-triggered group suffered from more severe pre-existing heart failure. However, rates of heart failure’s long term pharmacological treatments (beta blockers, ACE and ARN inhibitors, MRA) were equally distributed between groups.

Moreover, even if gathering all non-VA triggers in a single group was intentionally made to avoid selection bias, it could generate a misclassification risk since it includes a wide range of etiologies (ischemic, myocarditis, sepsis, etc.) whose prognosis is sometimes radically different, as previously shown (3). Further studies could target the prognosis of other frequent CS’ triggers.

Hence, the more frequent need for heart transplantation or VAD in the VA group without mortality difference suggest considering the occurrence of life-threatening VA as a pejorative turning point in heart failure, indicating a progression through the stages of disease severity. A retrospective monocentric study (14) directed on 222 patients (with 14 VT triggered CS) found similar results, emphasizing that even if VA can have a hemodynamic impact, it does not seem to increase early mortality. However, others reported that end-stage heart failure was the main cause of death after an electrical storm (20) which is consistent with our results considering all types of ventricular arrhythmia. That highlights that ventricular arrhythmia is a marker of advanced heart failure that could lead to discussion of advanced heart failure therapies like VAD and heart transplantation.

Other surveys focused on mortality prognosis factors in CS. First, the CardShock study (21) identified short-term mortality prognosis such as prior CABG, ACS etiology, confusion, previous myocardial infarction, blood lactate, LVEF, age and systolic blood pressure, which were all equally distributed between VA non-VA triggered CS in our study. Thereafter, the FAST-MI registry (8) revealed long term mortality prognosis such as age, diabetes mellitus or history of kidney disease, also fairly distributed between the two groups studied here.

Whether the ischemic nature of the underlying heart disease worsens the prognosis remains unclear. Indeed, while some studies found that non-ischemic CS was associated with higher mortality and use of catecholamines (9), other showed up to four times higher risk of death for ischemic heart disease (22, 23). These surveys considered CS regardless of the additional trigger and sometimes with differences in baseline characteristics between groups [such as BMI or sex ratio (23)]. In our study, when CS was triggered by VA, non-ischemic cardiomyopathy required more heart transplantation and VAD compared to ischemic cardiomyopathy, without all-cause mortality difference. In another study (24), early VT recurrence after ablation in non-ischemic cardiomyopathy resulted in higher risk for mortality or heart transplantation, urging to screening for mechanical circulatory support or heart transplantation, consistent with our results. However, several elements, such as more frequent previous ICD or even the more common use of betablockers could indicate that the group of non-ischemic VA-triggered CS was made up with more severe pre-existing heart failure, which may partly explain their poorer outcomes.

## Limitations

As previously described (3), the FRENSHOCK registry might be affected by selection bias related to non-consecutive inclusions or exclusion of the most severe cases. Moreover, the specific inclusion and exclusion criteria limit the applicability to all patients with CS.

Another limitation to mention is that SCAI SHOCK Stage Classification was not used for the group classification, given that this score was not yet available at the time of the study (25).

From available data, we defined the ischemic status as the presence of at least one culprit lesion on coronary angiography. Nevertheless, we were unable to separate STEMI, NSTEMI, and chronic coronary syndrome, whereas it is established that each of them carries different prognosis (8).

Even if major bias existed in some coding and biological data in this registry, additional analysis also revealed that, when considering acute ischemia as elevated troponin at the time of CS (thresholds at 10 μUI/L for standard troponin I, 200 μg/L for high sensitivity troponin I, and 2,000 ng/mL for high sensitivity troponin T) patients had a trend toward poorer outcome compared to VA triggered CS in stable ischemic heart disease, without reaching statistical significance. This seems surprising since VA in the setting of acute ischemia are not known to be a risk factor for the occurrence of late events. Confounding parameters probably explain this paradox, and especially elevated troponins could reflect more severity of CS and not only the cause.

The benefit of catheter ablation in electrical storm has already been demonstrated, proving its superiority to medical therapy in reducing arrhythmic burden (18, 19, 26–28). In our cohort, we don’t have enough data about VA to sort them between electrical storm and single isolated episodes. Further studies could specifically focus on detailed characteristics of ventricular arrhythmia and their impact on long-term outcomes.

In our study, 13 of the 94 VA-triggered CS had an ablation procedure. Such a low rate can be explained by different reasons. First, our study included many general hospitals in which facilities for carrying out an ablation are less developed. Moreover, the cohort was conducted in 2016, when this type of procedure was less common than today. Finally, we can assume that some patients did not necessarily need an ablation procedure, especially those with concomitant acute curable etiology (ACS, hypokalemia). Further studies could focus on the contribution of VA catheter ablation in advanced heart failure and the prospect of deferring transplantation or VAD in case of success.

## Conclusion

Ventricular arrhythmia is a common trigger of CS, which remains associated with high mortality outcomes comparable to non-VA-triggered CS. By contrast, it resulted in more heart transplantation and VAD at 1 year, especially in non-ischemic cardiomyopathy, suggesting the need for earlier evaluation by advanced heart failure specialized team for a possible indication of mechanical circulatory support or heart transplantation.

## Data availability statement

The raw data supporting the conclusions of this article will be made available by the authors, without undue reservation.

## Ethics statement

The studies involving human participants were reviewed and approved by CCTIRS (French Health Research Data Processing Advisory Committee) (no 15.897) and the CNIL (French Data Protection Agency) (no DR-2016-109). The patients/participants provided their written informed consent to participate in this study.

## Author contributions

FR, LB, GL, NL, BLe, GS, ME, PH, EB, EP, and CD: conception. MC, PM, and CD: methodology and initial draft of the manuscript. MC: statistics. PM and CD: supervision. CD: coordination. FR, ME, PH, EB, EP, and CD: sources and funding. All authors contributed to the data curation, investigation, manuscript review and editing, and validation.
