# Supplementary material for: One-year outcomes in cardiogenic shock triggered by ventricular arrhythmia: An analysis of the FRENSHOCK multicenter prospective registry
Source: Front Cardiovasc Med. 2023 Jan 26;10:1092904. doi: 10.3389/fcvm.2023.1092904 (PMC9909601; doi:10.3389/fcvm.2023.1092904)
Supplement: Supplementary file 1 [file Data_Sheet_1.pdf]

Supplemental material

One-year outcomes in cardiogenic shock triggered by ventricular arrhythmia: an analysis of the FRENSHOCK multicenter prospective registry, by Miloud Cherbi et al.

Table S1: Distribution of cardiogenic shock triggers between groups

Table S2: Clinical, echocardiographic, and biological parameters at 24h according to cardiogenic shock triggers

Table S3: Covariates used in the propensity score matching.

Table S4: Baseline characteristics at admission according to cardiogenic shock triggers (VA versus non-VA) after propensity score matching.

Table S5: Clinical, echocardiographic, and biological parameters at admission in the VA-triggered group according to cardiomyopathy**.**

Table S6: Clinical, echocardiographic, and biological parameters at 24h in the VA-triggered group according to cardiomyopathy**.**

Table S7: In-hospital management, short and long-term outcomes in the VA-triggered CS group according to the type of cardiomyopathy.

Table S8: Antiarrhythmic therapies in the VA group according to cardiomyopathy

Table S9: Short and long-term outcomes in the VA group according to troponin level.

Figure S1: All-cause mortality in the matched cohort according to a ventricular arrhythmia trigger

Figure S2: Mortality outcomes in the VA group according to various types of ischemic heart disease.

Figure S3: Mortality outcomes in the VA group according to troponin level

Table S1 Distribution of cardiogenic shock triggers between groups.

	**VA-triggered CS (n = 94)**	**Non-VA triggered CS (n = 675)**
Ischemic, n (%)	40 (42.6)	216 (32.0)
Mechanical complications, n (%)	5 (5.3)	19 (2.8)
Supra-ventricular arrhythmia, n (%)	0 (0)	100 (14.8)
Conduction disorder, n (%)	5 (5.3)	13 (1.9)
Infectious disease, n (%)	1 (1.1)	91 (13.5)
Non-compliance, n (%)	3 (3.2)	24 (3.6)
Iatrogenesis, n (%)	3 (3.2)	44 (6.5)

Underlying cardiopathy was considered ischemic in the presence of at least one culprit lesion hemodynamically significant on coronary angiography (stenosis or thrombosis).

CS = cardiogenic shock, VA = ventricular arrhythmia

Table S2 **Clinical, echocardiographic, and biological parameters at 24h according to cardiogenic shock triggers.**

	Overall population (n = 769)	VA-triggered CS (n = 94)	Non-VA triggered CS (n = 675)	p value*
Clinical presentation at 24h				
Heart rate, mean ± SD, bpm	94.7 ± 23.5 (n = 680)	91.0 ± 26.0 (n = 83)	95.3 ± 23.1 (n = 597)	0.08
SBP, mean ± SD, mmHg	105.1 ± 20.3 (n = 682)	103.9 ± 21.1 (n = 83)	105.2 ± 20.2 (n = 599)	0.49
DBP, mean ± SD, mmHg	61.8 ± 12.9 (n = 681)	60.5 ± 12.3 (n = 83)	61.9 ± 13.0 (n = 598)	0.34
MBP, mean ± SD, mmHg	75.4 ± 13.9 (n = 679)	74.2 ± 13.3 (n = 83)	75.5 ± 13.9 (n = 596)	0.33
Sinus rhythm, n (%)	376 (61.9) (n = 607)	42 (62.7) (n = 67)	334 (61.9) (n = 540)	1
Mottling, n (%)	115 (20.1) (n = 572)	14 (21.2) (n = 66)	101 (20.0) (n = 506)	0.94
Blood tests at 24h, median (IQR)				
Sodium, mmol/L	136 (133 - 140) (n = 688)	137 (134.5 - 140) (n = 83)	136 (133 - 139) (n = 605)	0.05
Creatinin, µmol/L	128 (92 - 186.8) (n = 686)	106 (84.5 - 168) (n = 83)	129 (94 - 189) (n = 603)	0.04
Bilirubin, mg/L	17 (10 - 29) (n = 416)	15.5 (11 - 26.5) (n = 57)	18 (10 - 29) (n = 359)	0.56
Hemoglobin, g/dL	12 (10 - 13.5) (n = 503)	12 (10 - 13.8) (n= 68)	12 (10 - 13.5) (n = 435)	0.59
Arterial blood lactates, mmol/L	2 (1.1 - 2.5) (n = 362)	1.9 (1 - 2.2) (n = 56)	2 (1.1 - 2.6) (n = 306)	0.27
ASAT, UI/L	113.5 (42 - 426) (n = 448)	127 (44 - 561) ± 857.0 (n = 57)	113 (42 - 419.5) (n = 391)	0.75
ALAT, UI/L	73 (30.25 - 248) (n = 454)	93 (41.5 - 279.5) ± 884.7 (n = 59)	70 (29 - 241.5) (n = 395)	0.24
PT, %	61 (39 - 75) (n = 634)	70 (45 - 84) (n = 79)	60 (38 - 73.5) (n = 555)	< 0.01
Nt-proBNP, pg/mL	9,292 (5,539.5 – 18,582.5) (n = 87)	6,640 (3,558 – 15,170) (n = 13)	9,371 (5,711.8 – 18,636.8) (n = 74)	0.36
BNP, pg/mL	1,254 (691 – 2,178) (n = 123)	14,09 (466.75 – 2,207) (n = 16)	1,191 (693.5 – 2,096.5) (n = 107)	0.95
CRP, mg/L	47.5 (25 - 118.25) (n = 236)	37 (15-88) (n = 25)	48 (27 - 121.5) (n = 211)	0.22
Echocardiography at 24h				
LVEF, mean ± SD, %	27.9 ± 13.4 (n = 500)	28.4 ± 13.7 (n = 61)	27.8 ± 13.3 (n = 439)	0.65
TAPSE, median (IQR), mm	14 (11 - 18) (n = 141)	14 (11.5 - 18.5) (n = 19)	14 (11 - 17) (n = 122)	0.70
PSVtdi, median (IQR), cm/s	10 (8 - 12) (n = 114)	10 (9 - 14) (n = 13)	10 (8 - 12) (n = 101)	0.25

*p value refers to the comparison between VA and non-VA triggered CS.

ALAT = alanine aminotransferase, ASAT = aspartate aminotransferase, BNP = Brain natriuretic peptide, CRP = C-reactive protein, CS = cardiogenic shock, DBP = diastolic blood pressure, IQR = interquartile range, MBP = mean blood pressure, Nt-proBNP = N-terminal-pro hormone BNP, LVEF = left ventricular ejection fraction, PSVtdi = peak systolic velocity tissue Doppler imaging, PT = prothrombin time, SBP = systolic blood pressure, SD = standard deviation, TAPSE = tricuspid annular plane systolic excursion, VA = ventricular arrhythmia

Table S3 Covariates used in the propensity score matching

	Before matching			After matching		
	**VA-triggered CS (n = 94)**	**Non-VA triggered CS (n = 675)**	**Standardized mean difference**	**VA-triggered CS (n = 94)**	**Non-VA triggered CS (n = 564)**	**Standardized mean difference**
Age, mean ± SD	64.1 ± 14.5	66.0 ± 14.9	- 0.1315	64.1 ± 14.5	65.1 ± 15.1	- 0.0691
Male, n (%)	549 (71.4)	487 (72.1)	- 0.1306	549 (71.4)	386 (68.4)	- 0.0524
LVEF ≤ 40%, n (%)	82 (87.2)	578 (85.6)	0.0481	82 (87.2)	487 (86.3)	0.0266
History of ischemic heart disease, n (%)	24 (25.5)	206 (30.5)	- 0.0888	24 (25.5)	169 (30.0)	- 0.0762
Previous ICD, n (%)	14 (14.9)	113 (16.7)	- 0.0519	14 (14.9)	89 (15.8)	- 0.0249

The propensity score for each patient was estimated using a multivariable logistic regression model including 5 covariates (sex, age, history of ischemic heart disease, LVEF ≤ 40% at admission and previous ICD) that were prognostically important for the outcome. Subjects were matched using greedy nearest neighbor matching with 6:1 ratio and a caliper distance of 0.2 times the SD of the logit of the propensity score.

CS = cardiogenic shock, ICD = implantable cardiac defibrillator, LVEF = left ventricular ejection fraction, SD = standard deviation, VA = ventricular arrhythmia

Table S4 **Baseline characteristics at admission according to cardiogenic shock triggers (VA versus non-VA) after propensity score matching**

	Overall population (n = 658)	VA-triggered CS (n = 94)	Non-VA triggered CS (n = 564)	p value*
Age, mean ± SD, years	65 ± 15	64.1 ± 14.5	65.1 ± 15.1	0.52
Male, n (%)	448 (68.1)	62 (66.0)	386 (68.4)	0.72
Body mass index, mean ± SD, kg/m²	25.7 ± 5.6 (n = 634)	25.8 ± 4.6 (n = 88)	25.7 ± 5.7 (n = 546)	0.37
Risk factors, n (%)				
Diabetes mellitus	178 (27.1) (n = 656)	23 (24.5)	155 (27.6) (n = 562)	0.62
Hypertension	298 (45.4) (n = 657)	38 (40.4)	260 (46.2) (n = 563)	0.35
Dyslipidemia	222 (33.8) (n = 657)	38 (40.4)	184 (32.7) (n = 563)	0.18
Current smoker	176 (27.8) (n = 632)	29 (31.9) (n = 91)	147 (27.2) (n = 541)	0.42
Medical history, n (%)				
Peripheral artery disease	76 (11.6) (n = 657)	8 (8.5)	68 (12.1) (n = 563)	0.41
Myocardial revascularization	163 (24.8) (n = 657)	21 (22.3)	142 (25.2) (n = 563)	0.64
Chronic kidney failure	134 (20.4) (n = 657)	13 (13.8)	121 (21.5) (n = 563)	0.12
ICD	103 (15.7)	14 (14.9)	89 (15.8)	0.95
COPD	40 (6.1) (n = 657)	4 (4.3)	36 (6.4) (n = 563)	0.57
Active cancer	44 (7) (n = 657)	5 (5.3)	39 (6.9) (n = 563)	0.72
Stroke	56 (9.9) (n = 657)	9 (9.6)	47 (8.3) (n = 563)	0.85
History of cardiac disease, n (%)				
All causes	354 (53.9) (n = 657)	47 (50.0)	307 (54.5) (n = 563)	0.48
Ischemic	193 (29.3)	24 (25.5)	169 (30)	0.45
Hypertrophic	9 (1.4) (n = 657)	1 (1.1)	8 (1.4) (n = 563)	1
Toxic	30 (4.6) (n = 657)	4 (4.3)	26 (4.6) (n = 563)	1
Idiopathic	70 (10.7) (n = 657)	12 (12.8)	58 (10.3) (n = 563)	0.59

Previous medications, n (%)				
Aspirin	243 (37) (n = 656)	37 (39.4)	206 (36.7) (n = 562)	0.7
P2Y12 inhibitors	102 (18.1) (n = 656)	20 (21.3)	82 (14.6) (n = 562)	0.13
Vitamin K antagonist	137 (20.9) (n = 656)	12 (12.8)	125 (22.2) (n = 562)	0.051
Direct oral anticoagulant	47 (8.4) (n = 656)	5 (5.3)	42 (7.5) (n = 562)	0.59
ACEi or ARB	237 (36.1) (n = 656)	33 (35.1)	204 (36.3) (n = 562)	0.92
Sacubitril/valsartan	13 (2.1) (n = 632)	1 (1.1) (n = 91)	12 (2.2) (n = 541)	0.7
Statins	229 (34.9) (n = 656)	38 (40.4)	191 (34) (n = 562)	0.27
Beta blockers	263 (40.1) (n = 656)	38 (40.4)	225 (40) (n = 562)	1
Loop diuretics	310 (47.3) (n = 656)	40 (42.6)	270 (48) (n = 562)	0.38
Aldosterone antagonist	88 (13.4) (n = 656)	10 (10.6)	78 (13.9) (n = 562)	0.49
Thiazide diuretics	34 (5.3) (n = 641)	6 (6.5) (n = 93)	28 (5.1) (n = 548)	0.62
Non-dihydropyridine CCB	10 (1.6) (n = 639)	0 (0) (n = 93)	10 (1.8) (n = 546)	0.37
Amiodarone	107 (16.7) (n = 640)	21 (22.6) (n = 93)	86 (15.7) (n = 547)	0.14
Other antiarrhythmic	26 (4.1) (n = 636)	7 (7.5) (n = 93)	19 (3.5) (n = 543)	0.09

*p value refers to the comparison between VA and non-VA triggered CS.

ACEi = angiotensin-converting enzyme inhibitor, ARB, angiotensin receptor blocker, CCB = calcium channel blocker, COPD = chronic obstructive pulmonary disease, CS = cardiogenic shock, ICD = implantable cardiac defibrillator, SD = standard deviation, VA = ventricular arrhythmia

Table S5 **Clinical, echocardiographic, and biological parameters at admission in the VA-triggered group according to cardiomyopathy.**

	**Ischemic VA-triggered CS (n = 40)**	**Non-ischemic VA triggered CS (n = 54)**	**p value**
Age, mean ± SD, years	66.2 ± 12.2	62.5 ± 15.9	0.54
Male, n (%)	26 (65)	36 (66.7)	1
Body mass index, mean ± SD, kg/m²	26.5 ± 4.6 (n = 37)	25.3 ± 4.7 (n = 51)	0.18
Risk factors, n (%)			
Diabetes mellitus	12 (30)	11 (20.4)	0.41
Hypertension	18 (45)	20 (37.0)	0.57
Dyslipidemia	15 (37.5)	23 (42.6)	0.78
Current smoker	15 (38.5) (n = 39)	14 (26.9) (n = 52)	0.35
Medical history, n (%)			
Peripheral artery disease	3 (7.5)	5 (9.3)	1
Myocardial revascularization	8 (20)	13 (24.1)	0.83
Chronic kidney failure	4 (10)	9 (16.7)	0.53
ICD	0 (0)	14 (25.9)	< 0.01
COPD	2 (5)	2 (3.7)	1
Active cancer	4 (10)	1 (1.9)	0.16
Stroke	2 (5)	7 (13.0)	0.29

Previous medications, n (%)			
Aspirin	17 (42.5)	20 (37.0)	0.75
P2Y12 inhibitors	9 (22.5)	11 (20.4)	1
Vitamin K antagonist	3 (7.5)	9 (16.7)	0.32
Direct oral anticoagulant	0 (0)	5 (9.3)	0.13
ACEi or ARBs	11 (27.5)	22 (40.7)	0.27
Sacubitril/valsartan	0 (0)	1 (2.0) (n = 51)	1
Statins	16 (40)	22 (40.7)	1
Beta blockers	9 (22.5)	29 (53.7)	< 0.01
Loop diuretics	13 (32.5)	27 (50)	0.14
Aldosterone antagonist	4 (10)	6 (11.1)	1
Thiazide diuretics	4 (10.3) (n = 39)	2 (3.7)	0.23
Non-dihydropyridine CCB	0 (0)	0 (0) (n = 53)	NA
Amiodarone	7 (17.5)	14 (26.4) (n = 53)	0.44
Other antiarrhythmic	3 (7.5)	4 (7.5) (n = 53)	1
Clinical presentation at admission			
Heart rate, mean ± SD, bpm	101.6 ± 36.0	102.3 ± 47.1	0.64
SBP, mean ± SD, mmHg	103.7 ± 22.2	95.1 ± 24.2	0.09
DBP, mean ± SD, mmHg	63.7 ± 16.3	59.3 ± 17.1	0.18
MBP, mean ± SD, mmHg	76.6 ± 17.8	70.8 ± 19.2	0.16
Sinus rhythm, n (%)	18 (45)	14 (25.9)	0.09
Mottling, n (%)	14 (38.9) (n = 36)	24 (54.6) (n = 44)	0.24
Cardiac arrest, n (%)	15 (37.5)	13 (24.1)	0.24
Blood tests at admission, median (IQR)			
Sodium, mmol/L	135 (132.5 – 139.5) (n = 39)	135 (133 – 138)	0.84
Creatinin, µmol/L	115 (82.5 – 138.5) (n = 39)	138 (97 – 202.75)	0.03
Bilirubin, mg/L	12 (7.5 – 15) (n = 35)	21 (12 – 29.75) (n = 42)	< 0.01
Hemoglobin, g/dL	13.2 (11.95 – 14.15) (n = 39)	13 (10.5 – 14) (n = 53)	0.22
Arterial blood lactates, mmol/L	2.88 (2 – 5.93) (n = 38)	2.59 (2 – 6.16) (n = 52)	0.99
ASAT, UI/L	185 (65.5 – 371.25) (n = 32)	96 (43 – 418.75) (n = 38)	0.42
ALAT, UI/L	87 (43 – 195) (n = 33)	97 (34.5 – 403.5) (n = 39)	0.71

PT, %	71 (55.3 – 86.8) (n = 38)	53.5 (32 – 73) (n = 52)	< 0.01
Nt-proBNP, pg/mL	1,520 (487.8 – 5,629.3) (n = 14)	6,787 (3,765 – 25,468.5) (n = 23)	0.046
BNP, pg/mL	476 (207.5 – 2,016) (n = 11)	776.5 (435.5 – 1,818) (n = 16)	0.73
CRP, mg/L	10 (4 – 40) (n = 21)	15.5 (5.75 – 46.5) (n = 24)	0.96
Baseline echocardiography			
LVEF, mean ± SD, %	23.2 ± 12.0 (n = 39)	25.3 ± 14.0	0.6
TAPSE, median (IQR), mm	15 (11.3 – 17.5) (n = 16)	13.5 (10 – 15.3) (n = 16)	0.44
PSVtdi, median (IQR), cm/s	9 (8 – 18) (n = 9)	8 (6 – 11.5) (n = 15)	0.23
Severe mitral regurgitation, n (%)	2 (5.4) (n = 37)	8 (16) (n = 50)	0.18
Severe aortic stenosis, n (%)	0 (0) (n = 39)	1 (1.9) (n = 53)	1
Severe aortic regurgitation, n (%)	1 (2.6) (n = 39)	1 (1.9) (n = 53)	1

ACEi = angiotensin-converting enzyme inhibitor, ALAT = alanine aminotransferase, ARB = angiotensin receptor blockers, ASAT = aspartate aminotransferase, BNP = Brain natriuretic peptide, CCB = calcium channel blocker, COPD = chronic obstructive pulmonary disease, CRP = C-reactive protein, CS = cardiogenic shock, DBP = diastolic blood pressure, ICD = implantable cardiac defibrillator, IQR = interquartile range, LVEF = left ventricular ejection fraction, Nt-proBNP = N-terminal-pro hormone BNP, PSVtdi = peak systolic velocity tissue Doppler imaging, PT = prothrombin time, SBP = systolic blood pressure, SD = standard deviation, TAPSE = tricuspid annular plane systolic excursion, VA = ventricular arrhythmia

Table S6 **Clinical, echocardiographic, and biological parameters at 24h in the VA-triggered group according to cardiomyopathy.**

	Ischemic VA-triggered CS (n = 40)	Non-ischemic VA-triggered CS (n = 54)	p value
Clinical presentation at 24h			
Heart rate, mean ± SD, bpm	92.3 ± 24.8 (n = 35)	90 ± 27.1 (n = 48)	0.7
SBP, mean ± SD, mmHg	102.8 ± 24.5 (n = 35)	104.6 ± 18.4 (n = 48)	0.8
DBP, mean ± SD, mmHg	58.1 ± 11.4 (n = 35)	62.2 ± 12.8 (n = 48)	0.21
MBP, mean ± SD, mmHg	73.2 ± 13.2 (n = 35)	75.0 ± 13.5 (n = 48)	0.91
Sinus rhythm, n (%)	19 (65.5) (n = 29)	23 (60.5) (n = 38)	0.87
Mottling, n (%)	5 (17.2) (n = 29)	9 (24.3) (n = 37)	0.69
Blood tests at 24h, median (IQR)			
Sodium, mmol/L	138 (135 – 140.5) (n = 35)	137 (133 – 140) (n = 48)	0.42
Creatinin, µmol/L	103 (81 – 157.5) (n = 35)	113 (90.25 – 195) (n = 48)	0.32
Bilirubin, mg/L	13.6 (9.25 – 16.08) (n = 26)	22 (12 – 34.5) (n = 31)	< 0.01
Haemoglobin, g/dL	12.1 (11 – 13.13) (n = 32)	12 (10 – 14) (n = 36)	0.96
Arterial blood lactates, mmol/L	1.9 (1.1 – 2.55) (n = 27)	1.93 (1 – 2) (n = 29)	0.65
ASAT, UI/L	307 (66 – 676) (n = 29)	68.5 (32.75 – 319.5) (n = 28)	0.06
ALAT, UI/L	104 (54 – 244) (n = 29)	76.5 (37.8 – 421.5) (n = 30)	0.84
PT, %	73.5 (62 – 89.5) (n = 34)	61 (42 – 73) (n = 45)	< 0.01
Nt-proBNP, pg/mL	5,070.5 (4,077.8 – 8,123) (n = 4)	10,271 (3,558 – 23,000) (n = 9)	0.6
BNP, pg/mL	1,215 (436.3 – 2,087) (n = 10)	1,460 (1,274.8 – 2,071.3) (n = 6)	0.87
CRP, mg/L	52 (31.8 – 81.3) (n = 14)	16 (8.5 – 103) (n = 11)	0.25
Echocardiography at 24h			
LVEF, mean ± SD, %	27.6 ± 11.6 (n = 28)	29.0 ± 15.4 (n = 33)	0.93
TAPSE, median (IQR), mm	13 (10.5 – 18.5) (n = 7)	14.5 (12 – 17.5) (n = 12)	0.69
PSVtdi, median (IQR), cm/s	12 (10 – 17.8) (n = 6)	10 (8.5 – 11) (n = 7)	0.31

ALAT = alanine aminotransferase, ASAT = aspartate aminotransferase, BNP = Brain natriuretic peptid, CRP = C-reactive protein, CS = cardiogenic shock, DBP = diastolic blood pressure, IQR = interquartile range, LVEF = left ventricular ejection fraction, Nt-proBNP = N-terminal-pro hormone BNP, PSVtdi = peak systolic velocity tissue Doppler imaging, PT = prothrombin time, SBP = systolic blood pressure, SD = standard deviation, TAPSE = tricuspid annular plane systolic excursion, VA = ventricular arrhythmia

Table S7 **In-hospital management, short and long-term outcomes in the VA-triggered CS group according to the type of cardiomyopathy.**

	Ischemic VA-triggered CS (n = 40)	Non-ischemic VA triggered CS (n = 54)	p value
Medications used, n (%)			
Dobutamine or norepinephrine or levosimendan	37 (92.5)	44 (81.5)	0.22
Dobutamine	34 (85)	38 (70.4)	0.16
Norepinephrine	25 (62.5)	32 (59.3)	0.92
Levosimendan	2 (5)	3 (5.6)	1
Respiratory support, n (%)			
Non-invasive	10 (25)	14 (25.9)	1
Invasive	19 (47.5)	29 (53.7)	0.70
Short-term mechanical circulatory support, n (%)			
IABP	6 (15)	1 (1.85)	0.04
Impella	1 (2.5)	1 (1.85)	1
ECLS	7 (17.5)	9 (16.7)	1
Renal replacement therapy, n (%)	2 (5)	11 (20.4)	0.07
LVEF at discharge, mean ± SD	34.5 ± 9.0 (n = 20)	41.5 ± 17.3 (n = 32)	0.09
Mortality, n (%)			
1 month	12 (30)	13 (24.1)	0.68
1 year	17 (42.5)	23 (42.6)	1
Rehospitalizations at 1 year, n (%)	17 (45.9) (n = 37)	24 (49.0) (n = 49)	0.95
Mortality or rehospitalizations at 1 year, n (%)	31 (83.8) (n = 37)	40 (81.6) (n = 49)	1
Heart transplantation or VAD at 1 year, n (%)	2 (5)	14 (25.9)	0.02
Mortality or heart transplantation or VAD at 1 year, n (%)	19 (47.5)	34 (63.0)	0.20

CS = cardiogenic shock, ECLS = extracorporeal life support, IABP = intra-aortic balloon pump, LVEF = left ventricle ejection fraction, SD = standard deviation, VA = ventricular arrhythmia, VAD = ventricular assistant device

Table S8 **Antiarrhythmic therapies in the VA group according to cardiomyopathy.**

	Ischemic VA-triggered CS (n = 40)	Non-ischemic VA-triggered CS (n = 54)	p value
Betablockers, n (%)			
Initial care	9 (2.25)	29 (53.7)	< 0.01
24h	5 (14.3) (n = 35)	10 (20.8) (n = 48)	0.63
Discharge	19 (70.4) (n = 27)	18 (47.4) (n = 38)	0.11
1 year	17 (81.0) (n = 21)	12 (57.1) (n = 21)	0.18
Non-dihydropyridine CCB, n (%)			
Initial care	0 (0)	0 (0) (n = 53)	NA
24h	1 (2.94) (n = 34)	0 (0) (n = 45)	0.43
Discharge	0 (0) (n = 27)	0 (0) (n = 35)	NA
1 year	0 (0) (n = 20)	0 (0) (n = 20)	NA
Amiodarone, n (%)			
Initial care	7 (17.5)	14 (26.4) (n = 53)	0.44
24h	14 (41.2) (n = 34)	28 (59.6) (n = 47)	0.16
Discharge	5 (19.2) (n = 26)	9 (25) (n = 36)	0.82
1 year	1 (5) (n = 20)	2 (10) (n = 20)	1
Other anti-arrhythmic, n (%)			
Initial care	3 (7.5)	4 (7.6) (n = 53)	1
24h	4 (11.8) (n = 34)	8 (17.4) (n = 46)	0.7
Discharge	3 (11.1) (n = 27)	3 (8.3) (n = 36)	1
1 year	2 (10) (n = 20)	2 (10) (n = 20)	1
ICD implantation, n (%)	2 (5)	9 (17.0) (n = 53)	0.11
VA catheter ablation, n (%)	4 (10)	9 (17.0) (n = 53)	0.51

CCB = calcium channel blocker, CS = cardiogenic shock, ICD = Implantable cardioverter-defibrillator, VA = ventricular tachycardia

Table S9 **Short and long-term outcomes in the VA group according to troponin level.**

	VA-triggered CS with high troponin (n = 13)	VA-triggered CS with low troponin (n = 81)	p value
Mortality, n (%)			
1 month	4 (30.8)	21 (25.9)	0.74
1 year	6 (46.2)	34 (42)	1
Rehospitalizations at 1 year, n (%)	6 (46.2)	35 (47.9) (n = 73)	1
Mortality or rehospitalizations at 1 year, n (%)	11 (84.6)	60 (82.2) (n = 73)	1
Heart transplantation or VAD at 1 year, n (%)	1 (7.7)	15 (18.5)	0.46
Mortality or heart transplantation or VAD at 1 year, n (%)	7 (53.8)	46 (56.8)	1

High troponin was defined at the time of CS with a threshold of 10 µUI/L for standard troponin I, of 200 µg/L for high sensitivity troponin I and of 2000 ng/mL for high sensitivity troponin T. CS = cardiogenic shock, VA = ventricular arrhythmia, VAD = ventricular assist device

FIGURE S1: All-cause mortality in the matched cohort according to a ventricular arrhythmia trigger

Matched VA-triggered CS — Matched non-VA-triggered CS

A. 1 year mortality **B. 1 month mortality**

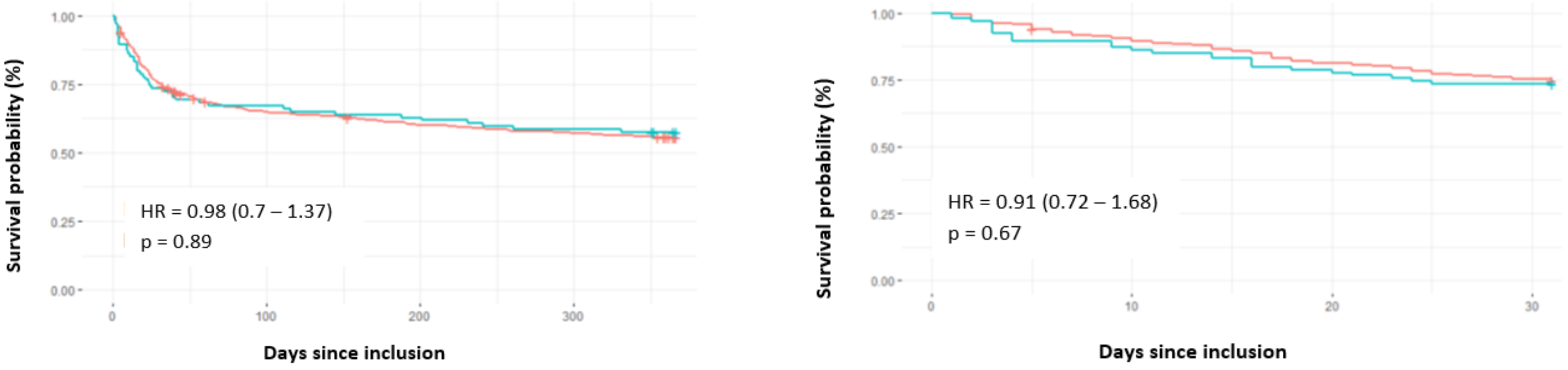

A. 1 year mortality

Matched VA-triggered CS	94	63	59	55
Matched Non-VA triggered CS	564	358	332	315

B. 1 month mortality

Matched VA-triggered CS	94	82	74	69
Matched Non-VA triggered CS	564	510	458	423

Panel A represents one-year overall mortality. Panel B focus on one-month mortality. The cumulative incidences of one-year and one-month mortality were estimated with the use of the Kaplan–Meier method; hazard ratios and 95% confidence intervals were estimated with the use of Cox regression models.

CS = cardiogenic shock, HR = hazard ratio, VA = ventricular arrhythmia

FIGURE S2: Mortality outcomes in the VA group according to various types of ischemic heart disease.

Acute ischemic VA-triggered CS — VA-triggered CS with stable ischemic heart disease

A. 1 year mortality

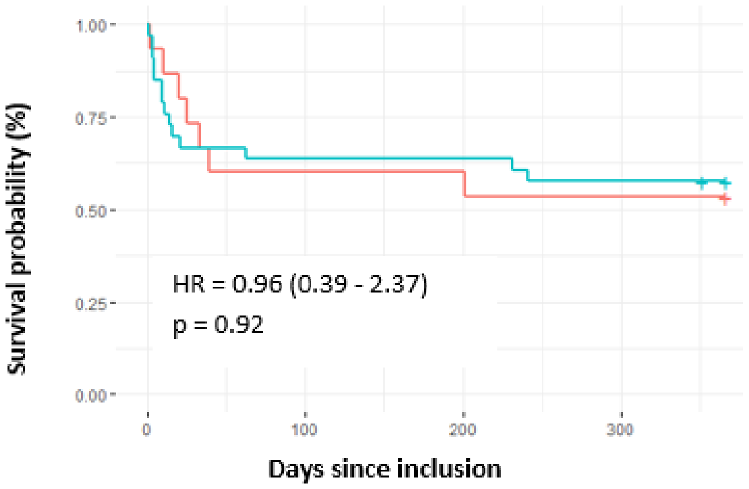

Number at risk				
Acute ischemic VA-triggered CS	33	21	21	19
VA-triggered CS with stable ischemic heart disease	15	9	9	8

B. 1 month mortality

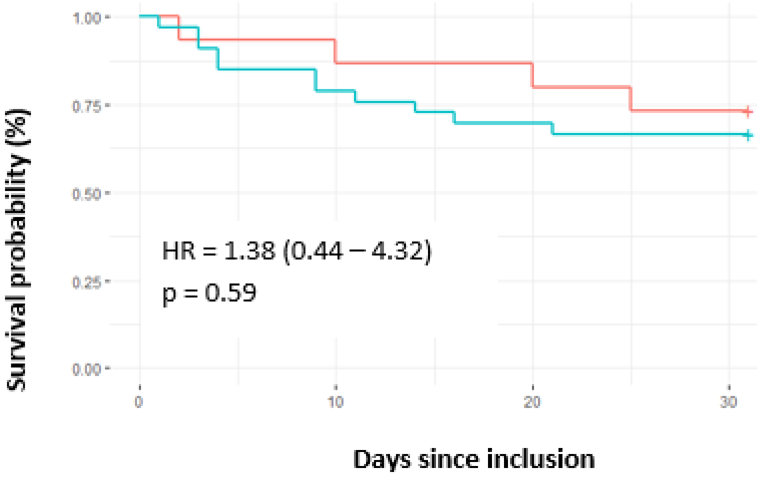

Number at risk				
Acute ischemic VA-triggered CS	33	26	23	22
VA-triggered CS with stable ischemic heart disease	15	14	13	11

Panel A represents one-year overall mortality. Panel B focus on one-month mortality. The cumulative incidences of one-year and one-month mortality were estimated with the use of the Kaplan–Meier method; hazard ratios and 95% confidence intervals were estimated with the use of Cox regression models. Underlying cardiopathy was considered acute ischemic when indicated as such by managing physician. Ischemic heart disease was considered stable in case of previously known ischemic cardiomyopathy without new lesion on repeated coronary angiogram.

CS = cardiogenic shock, HR = hazard ratio, VA = ventricular arrhythmia

FIGURE S3: Mortality outcomes in the VA group according to troponin level

VA-triggered CS with high troponin — VA-triggered CS with low troponin

A. 1 year mortality **B. 1 month mortality**

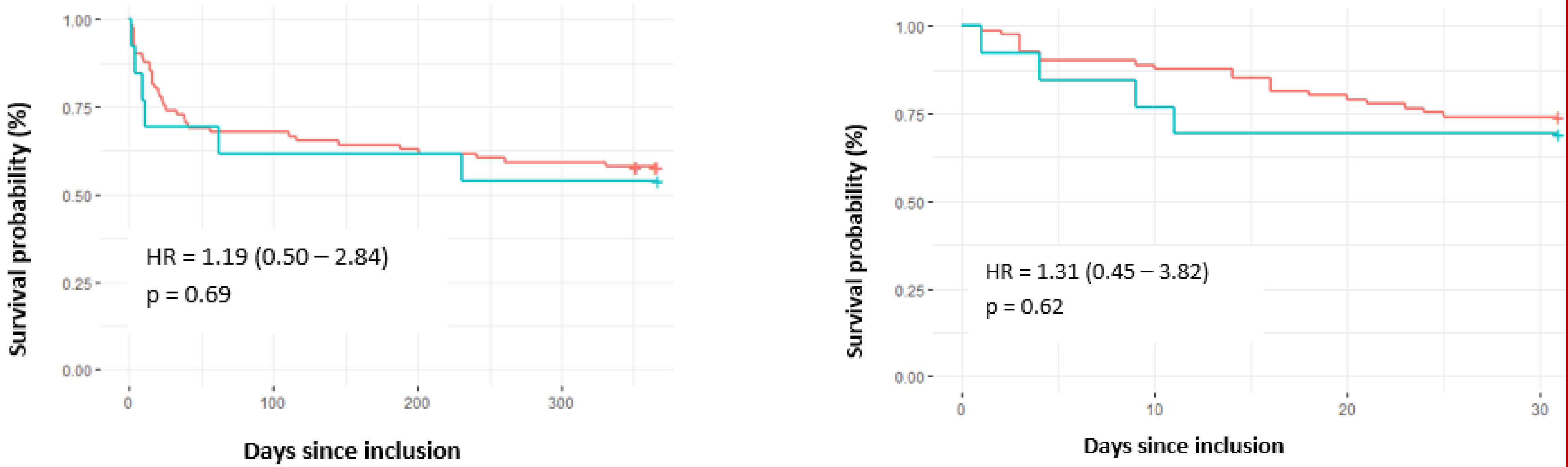

A.

VA-triggered CS with high troponin	13	8	8	7
VA-triggered CS without high troponin	81	55	51	48

B.

VA-triggered CS with high troponin level	13	10	9	9
VA-triggered CS without high troponin level	81	72	65	60

Panel A represents one-year overall mortality. Panel B focus on one-month mortality. The cumulative incidences of one-year and one-month mortality were estimated with the use of the Kaplan–Meier method; hazard ratios and 95% confidence intervals were estimated with the use of Cox regression models. The threshold for high troponin was set at 10 µUI/L for standard troponin I, 200 µg/L for high sensitivity troponin I, and 2000 ng/mL for high sensitivity troponin T.

CS = cardiogenic shock, HR = hazard ratio, VA = ventricular arrhythmia
